# A cuproptosis-related gene signature and associated regulatory axis in stomach adenocarcinoma based on bioinformatics analysis

**DOI:** 10.1097/MD.0000000000034230

**Published:** 2023-07-28

**Authors:** Dongxiao Ding, Dianqian Wang, Yunsheng Qin

**Affiliations:** a Health Science Center, Ningbo University, Ningbo, Zhejiang, China; b Department of Thoracic Surgery, The People’s Hospital of Beilun District, Ningbo, Zhejiang, China; c Department of Hepatobiliary and Pancreatic Surgery, First Affiliated Hospital, School of Medicine, Zhejiang University, Hangzhou, Zhejiang, China; d Department of Hepatobiliary Surgery, The People’s Hospital of Beilun District, Ningbo, Zhejiang, China.

**Keywords:** cuproptosis, FDX1, MALAT1, prognostic signature, Stomach adenocarcinoma

## Abstract

Stomach adenocarcinoma (STAD) is a highly aggressive and extremely heterogeneous gastric cancer characterized by high morbidity and mortality. Cuproptosis, a copper (Cu)-triggered modality of mitochondrial cell death, could regulate tumor proliferation and metastasis. Least absolute shrinkage and selection operator cox regression analysis was constructed to develop a prognostic cuproptosis-related signature. A lncRNA-miRNA-mRNA regulatory axis was performed to explore cuproptosis-related mechanism for STAD. The expression of FDX1, LIPT1, DLD, DLAT, PDHA1, PDHB, MTF1, GLS, and CDKN2A was upregulated in STAD versus normal tissue. We also summarized single nucleotide variants and copy number variation landscape of cuproptosis-related gene in STAD. Further analysis demonstrated that STAD patients with high expression of CDKN2A, DLD, GLS, and MTF1 and low expression of DLAT, FDX1, PDHA1 and PDHB had a poor overall survival (OS) and post progression survival (PPS) rate. By performing least absolute shrinkage and selection operator cox regression analysis, we constructed a cuproptosis-related prognostic signature for STAD. Further analysis demonstrated a significant correlation between FDX1 expression and immune cell infiltration, tumor mutational burden (TMB) score, microsatellite instability (MSI) score and drug sensitivity. Univariate and multivariate analysis indicated FDX1 expression and clinical stage as independent factors affecting the prognosis of STAD patients. We also identified a lncRNA MALAT1/miR-328-3p/FDX1 regulatory axis for STAD. Multi-omics approaches were performed to develop a cuproptosis-related signature with 2 genes (FDX1 and MTF1) for STAD. We also identified a lncRNA MALAT1/miR-328-3p/FDX1 regulatory axis for STAD.

## 1. Introduction

Stomach adenocarcinoma (STAD) is a highly aggressive and extremely heterogeneous gastric cancer characterized by high morbidity and mortality.^[1]^ Every year, an estimated of 1 million people are initially diagnosed with gastric cancer and 760,000 patients die from this disease globally.^[2]^ Despite multidisciplinary approach could be afford to STAD patients, the prognosis of STAD patients was unacceptable.^[3]^ There are little effectively predictive markers for the prognosis of STAD clinically.^[4]^ The specific mechanism of STAD was not evaluated, though some risk factors were identified for STAD. These grim data highlight the urgent need for prognostic biomarkers and molecular mechanism of STAD.

Cuproptosis is a copper (Cu)-triggered modality of mitochondrial cell death different from apoptosis, ferroptosis, and necroptosis.^[5]^ Increasing evidence highlights the importance of Cu in many biological processes, such as mitochondrial respiration and detoxification processes.^[6]^ Moreover, cuproptosis-related genes could regulate tumor cell proliferation, invasion, and metastasis.^[6]^ Given that cuproptosis is different from apoptosis, ferroptosis, and necroptosis,^[7,8]^ understanding how cuproptosis is initiated and propagated may help identify cuproptosis-associated therapeutic interventions and possible combination treatments for human cancer.^[9]^ A previous study identified a novel cuproptosis-related prognostic signature for clear cell renal cell carcinoma (RCC).^[10]^ Another study showed that cuproptosis-related lncRNA signature could predict prognosis in hepatocellular carcinoma.^[11]^ Huang et al also developed cuproptosis-related subtypes and a prognostic signature in colorectal cancer.^[12]^ Based on the current literatures, there are few studies about the significance of cuproptosis-related genes in the progression and prognosis in STAD.

With the development of the Cancer Genome Atlas (TCGA, https://portal.gdc.cancer.gov/) and International Cancer Genome Consortium (ICGC, https://dcc.icgc.org/), database mining was suggested as one of the fastest and most effective approaches to identify biomarkers for the prognosis of human cancer. Through this research, we aimed to investigate the prognostic value of cuproptosis-related genes and their associated mechanism in STAD by mining TCGA and ICGC.

## 2. Materials and methods

### 2.1. Dataset downloading and gene expression analysis

A total 10 cuproptosis-related genes (FDX1, LIAS, LIPT1, DLD, DLAT, PDHA1, PDHB, MTF1, GLS, and CDKN2A) were included in our study based on previous study.^[1]^ After downloading the RNA-seq data of STAD from TCGA (https://portal.gdc.cancer.gov/) and ICGC (https://dcc.icgc.org/) database, we changed gene expression into transcripts per kilobase million value for expression normalization. R (version 4.0.3) was used to performed analysis and generate images with corresponding package. Student *t* test was utilized for gene expression analysis of cuproptosis-related genes in STAD.

### 2.2. Single nucleotide variants (SNV) and copy number variation (CNV) landscape, and functional enrichment pathway analysis

After downloading the cuproptosis-related SNV and CNV data in STAD from TCGA database, the landscape was visualized using “maftools” package in R software. Various types of SNV were included in our study (Missense_Mutation, Nonsense_Mutation, Frame_Shift_Ins, Splice_Site, Frame_Shift_Del, In_Frame_Del, In_Frame_Ins). CNV data was processed through GISTIC2.0 and it could identify significantly altered regions of amplification or deletion across sets of patients. “clusterProfiler” package was used to conducted gene ontology (GO) analysis and Kyoto encyclopedia of genes and genomes (KEGG) pathways analysis.

### 2.3. Construction of cuproptosis-related prognostic gene signature

To identify prognostic biomarkers, we then performed overall survival (OS) analysis and post progression survival (PPS) using differently expressed cuproptosis-related genes and log-rank test was utilized to calculate the *P* values, hazard ratio (HR) and 95% confidence interval. This was followed by least absolute shrinkage and selection operator cox regression analysis for the construction of a cuproptosis-related prognostic gene signature for STAD with prognostic biomarkers identified above. In the signature, the risk score of STAD patients was calculated by a computational equation (sum of coefficients × necroptosis-related gene expression). And STAD patients were divided into high-risk and low risk group with the median value as cutoff value. Kaplan–Meier method was applied to draw the OS curve and ROC analysis was performed to evaluate area under the curve (AUC). Moreover, ICGC dataset was used to further analyze this signature.

### 2.4. Immune infiltration and drug sensitivity analysis

TIMER (https://cistrome.shinyapps.io/timer/) is a portal for systematical analysis of tumor-infiltrating immune cells.^[13]^ “Gene” module of TIMER allows analysis about the correlation between cuproptosis-related prognostic signature and immune infiltration level in STAD with Pearson correlation test. We collected the IC50 of 481 small molecules in 1001 cell lines, and its corresponding cuproptosis-related prognostic signature gene expression from the Cancer of Therapeutics Response Portal (CTRP, http://portals.broadinstitute.org/ctrp.v2.1/). The cuproptosis-related prognostic signature gene expression data and drug sensitivity data were merged. Pearson correlation analysis was performed to get the correlation between cuproptosis-related prognostic signature and drug IC50. *P* value was adjusted by FDR. Moreover, correlation analysis between cuproptosis-related prognostic gene and Tumor Mutational Burden (TMB)/Microsatellite Instability (MSI) in TCGA was analyzed with the Spearman correlation test.

### 2.5. lncRNA-miRNA-mRNA regulatory axis analysis

In order to predict the miRNA targets of cuproptosis-related prognostic gene, we used 3 miRNA target predicting databases, including miRDB (http://mirdb.org/), StarBase (http://starbase.sysu.edu.cn/), and miRWalk (http://mirwalk.umm.uni-heidelberg.de/). To predict the lncRNA targets interacting with miRNA, we used 2 lncRNA databases (StarBase (http://starbase.sysu.edu.cn/) and LncBase module of DIANA tool (https://dianalab.e-ce.uth.gr/html/diana/web/index.php?r=tarbasev8)). The expression and prognostic value of miRNA and lncRNA target was evaluated as described above.

## 3. Results

### 3.1. The expression and mutation landscape of cuproptosis-related genes in STAD

**Figure 1.**
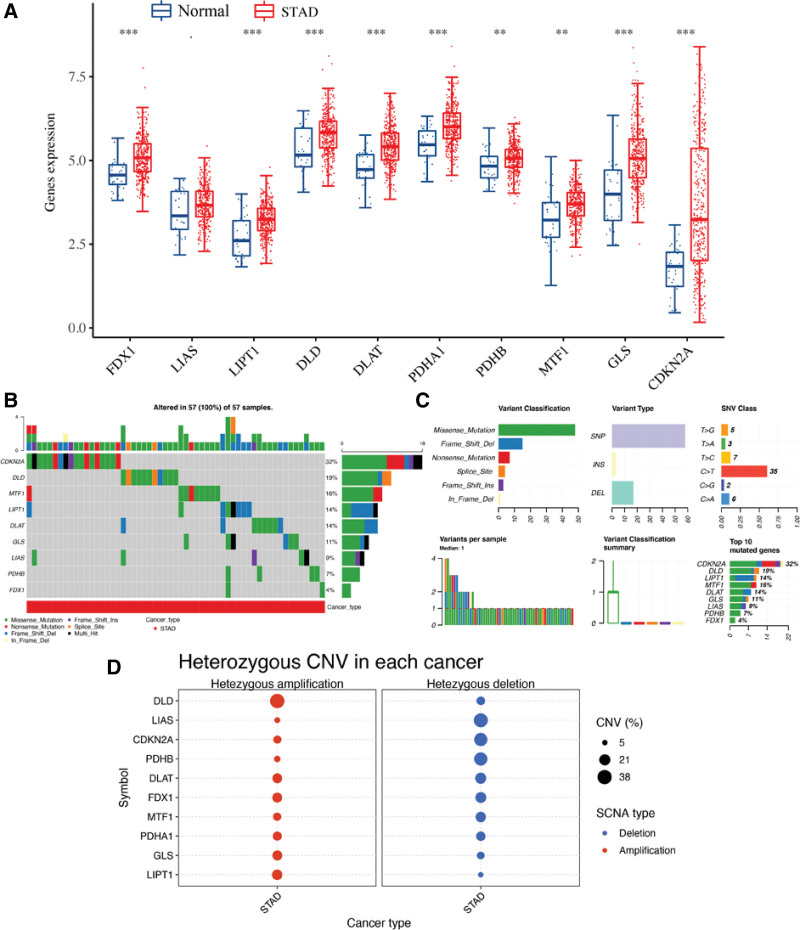
The expression, CNV and SNV landscape of cuproptosis-related genes in STAD. (A) The mRNA level of cuproptosis-related gene in STAD versus normal tissues in TCGA STAD dataset. (B and C) SNV landscape of cuproptosis-related gene in STAD. (D) CNV landscape of cuproptosis-related gene in STAD. *P < .05; **P < .01; ***P < .001; -*P* > .05. CNV = copy number variation, STAD = stomach adenocarcinoma, SNV = single nucleotide variants, TCGA = The Cancer Genome Atlas.

Among these 10 cuproptosis-related genes in STAD, 9 were altered in gene expression in STAD (Fig. 1A). The result revealed that the expression of FDX1, LIPT1, DLD, DLAT, PDHA1, PDHB, MTF1, GLS and CDKN2A was upregulated in STAD versus normal tissue in TCGA STAD dataset (Fig. 1A, all *P* < .01). The genetic mutation landscape of cuproptosis-related genes in STAD was shown in Figure 1B and C, revealing 32% of STAD samples with CDKN2A genetic mutation, followed by DLD (19%), MTF1(16%), LIPT1(14%), and DLAT (14%) (Fig. 1B). Missense mutation ranked the most common variant classification (Fig. 1C). And SNP and C > T were the most common variant type and SNV class (Fig. 1C). In CNV analysis, the data revealed that DLD had a significant homozygous amplification while LIAS, CDKN2A and PDHB had a widespread homozygous deletion (Fig. 1D).

### 3.2. The enrichment items in GO and KEGG pathway analysis

**Figure 2.**
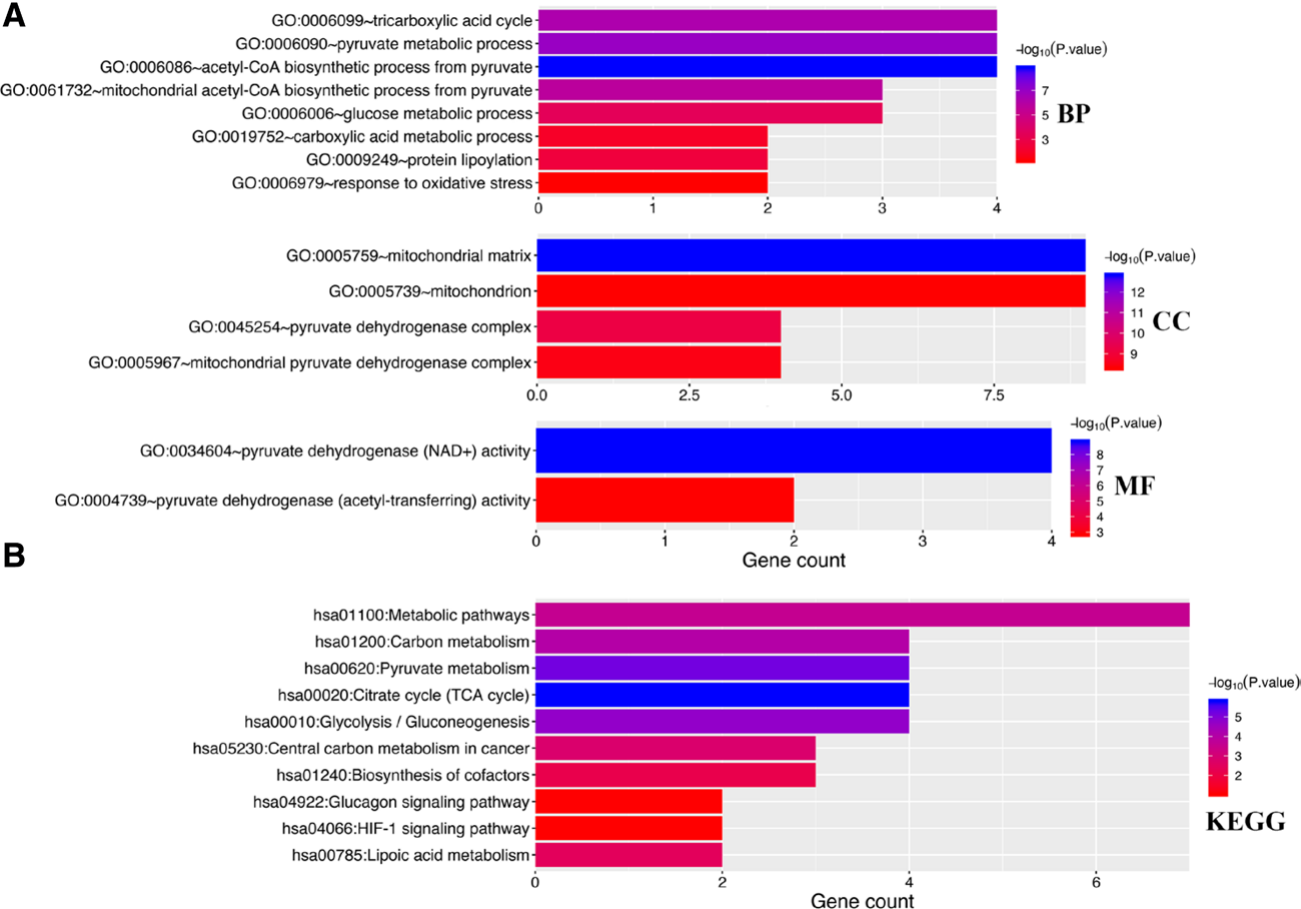
The enriched items in functional enrichment analysis. (A) The enriched items in gene ontology analysis. (B) The enriched items in Kyoto Encyclopedia of Genes and Genomes pathways analysis. BP = biological process, CC = cellular component, MF = molecular function.

As shown in Figure 2A, GO analysis demonstrated that cuproptosis-related genes were mainly associated with tricarboxyic acid cycle, pyruvate metabolic process, glucose metabolic process, mitochondrial matrix, NAD + activity, acetyl-transferring activity. Moreover, KEGG pathway analysis demonstrated that cuproptosis-related genes were mainly associated with metabolic pathways, carbon metabolism, TCA cycle, glucagon signaling pathway, HIF-1 signaling pathway, and lipoic acid metabolism (Fig. 2B).

### 3.3. Development of a cuproptosis-related prognostic signature in STAD

**Figure 3.**
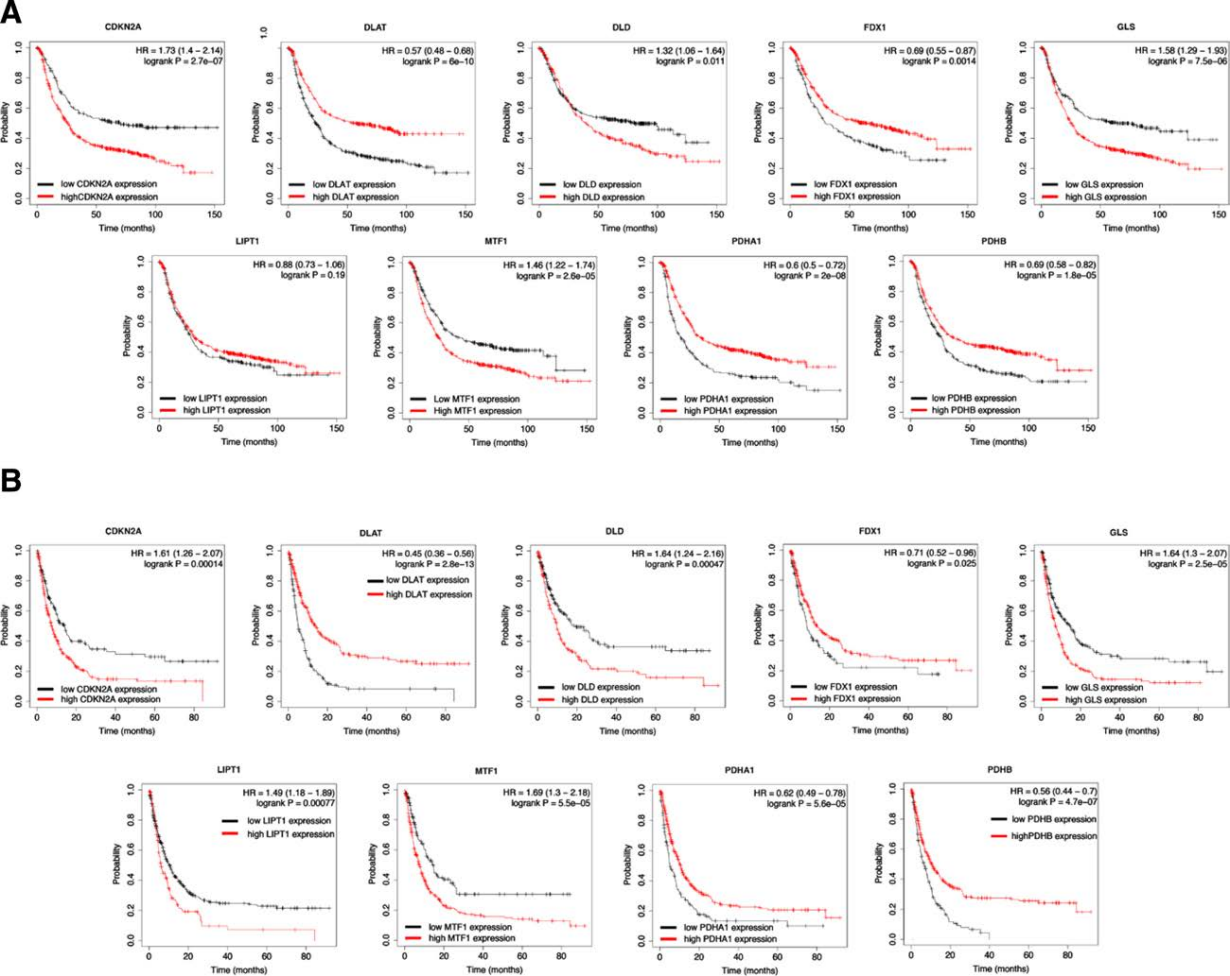
The prognostic significance of cuproptosis-related genes in STAD. (A) The overall survival curve in STAD patients with high and low expression of cuproptosis-related genes. (B) The recurrence-free survival curve in STAD patients with high and low expression of cuproptosis-related genes. STAD = stomach adenocarcinoma.

**Figure 4.**
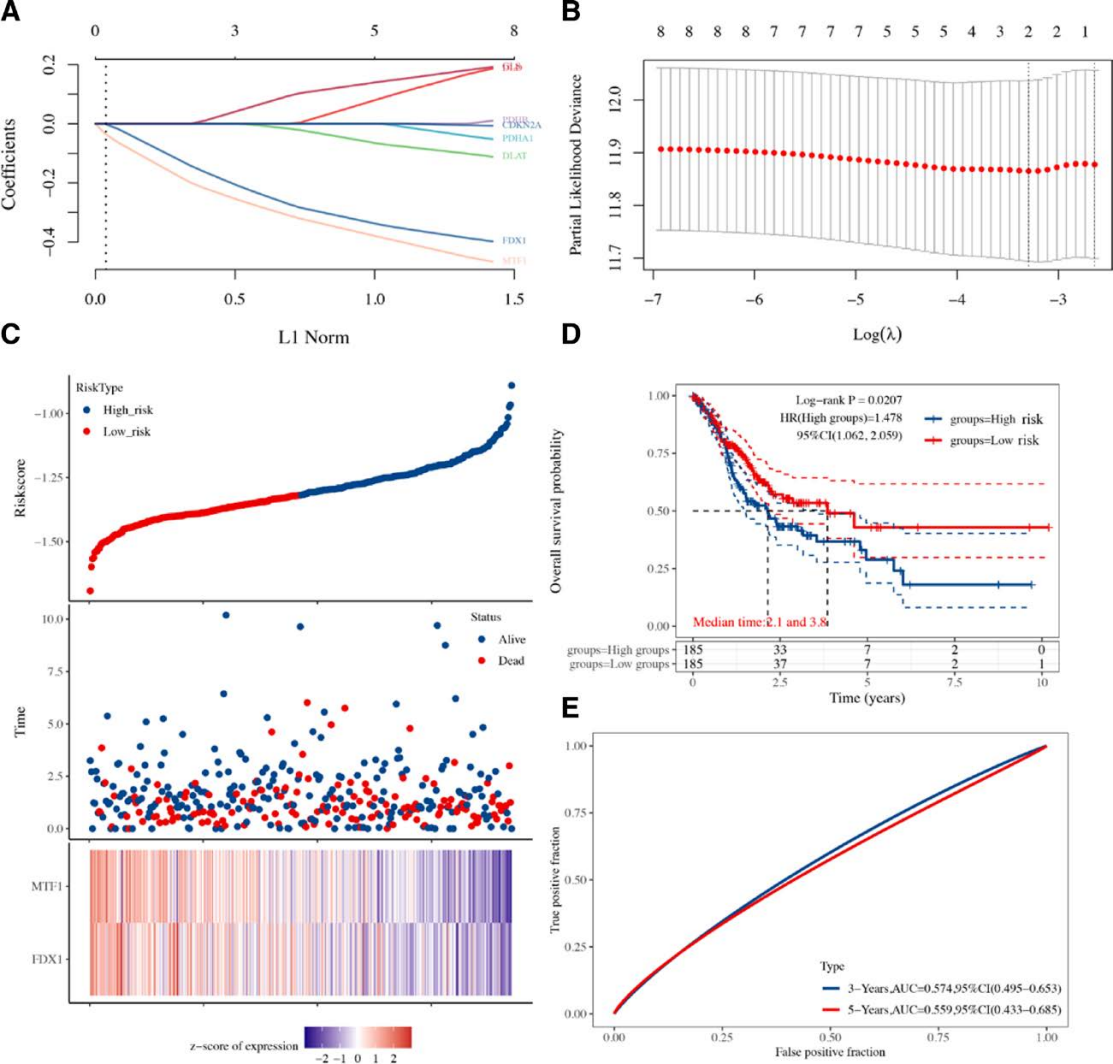
Cuproptosis-related prognostic signature for STAD in TCGA cohort. (A and B) The coefficient and partial likelihood deviance of prognostic signature. (C) The riskscore distribution, patient survival status and gene expression profile of prognostic signature. (D and E) The survival curve of STAD patients with high/low riskscore and ROC curve of prognostic signature. STAD = stomach adenocarcinoma, TCGA = The Cancer Genome Atlas.

**Figure 5.**
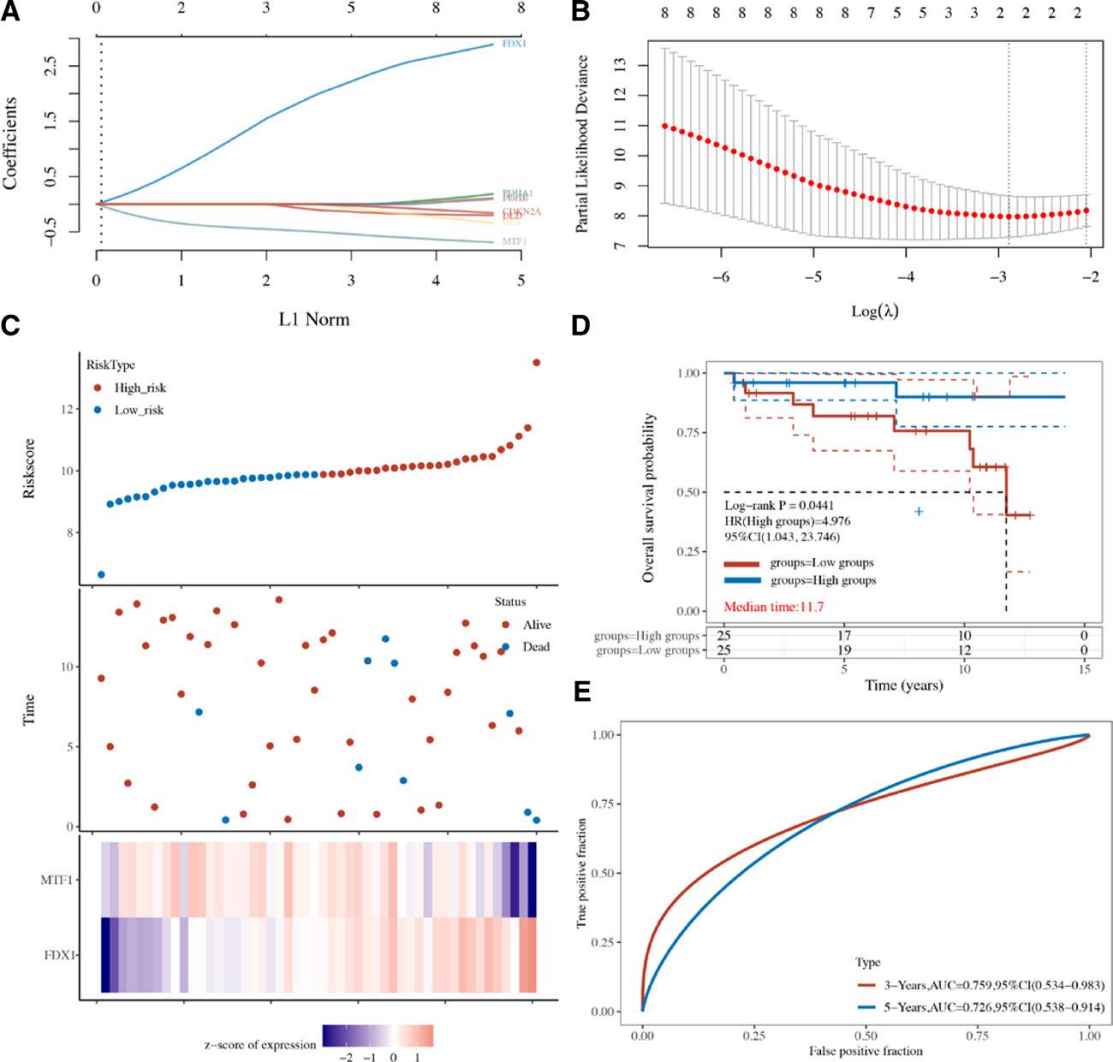
Cuproptosis-related prognostic signature for STAD in ICGC cohort. (A and B) The coefficient and partial likelihood deviance of prognostic signature. (C) The riskscore distribution, patient survival status and gene expression profile of prognostic signature. (D and E) The survival curve of STAD patients with high/low riskscore and ROC curve of prognostic signature. ICGC = International Cancer Genome Consortium, STAD = stomach adenocarcinoma.

We then performed OS analysis and PPS analysis to identify prognostic biomarkers for STAD. The data revealed that STAD patients with high level of CDKN2A (HR = 1.73, *P* = 2.7e^-7^), DLD (HR = 1.32, *P* = .011), GLS (HR = 1.58, *P* = 7.5e^-6^), and MTF1 (HR = 1.46, *P* = 2.6e^-5^) had a poor OS rate (Fig. 3A). The data also suggested a poor OS rate in STAD patients with low level of DLAT (HR = 0.57, *P* = 6e^-10^), FDX1 (HR = 0.69, *P* = .0014), PDHA1(HR = 0.6, *P* = 2e^-8^), and PDHB (HR = 0.69, *P* = 1.8e^-5^) (Fig. 3A). In PPS analysis, the data revealed that STAD patients with high level of CDKN2A (HR = 1.61, *P* = .00014), DLD (HR = 1.64, *P* = .00047), GLS (HR = 1.64, *P* = 2.5e^-5^), LIPT1 (HR = 1.49, *P* = .00077) and MTF1 (HR = 1.69, *P* = 5.5e^-5^) had a poor PPS rate (Fig. 3B). The data also suggested a poor PPS rate in STAD patients with low level of DLAT (HR = 0.45, *P* = 2.8e^-13^), FDX1 (HR = 0.71, *P* = .025), PDHA1(HR = 0.62, *P* = 5.6e^-5^), and PDHB (HR = 0.56, *P* = 4.6e^-7^) (Fig. 3B). This evidence suggested CDKN2A, DLAT, DLD, FDX1, GLS, MTF1, PDHA1, and PDHB as potential biomarkers for STAD. Based on these potential prognosis biomarkers, we performed least absolute shrinkage and selection operator cox regression analysis and a cuproptosis-related prognostic signature containing MTF1 and FDX1 was constructed for STAD. Figure 4A and B revealed the coefficient and partial likelihood deviance of cuproptosis-related prognostic signature in TCGA cohort. Figure 4C showed the risk score, survival status, and gene expression of prognostic signature. In prognosis analysis, TCGA STAD patients with high-risk score had a poor OS rate (Fig. 4D, *P* = .00207, HR = 1.48) with an AUC of 0.574 and 0.559 in a 3-year and 5-year ROC curve (Fig. 4E). We then verify prognostic signature using ICGC dataset. The coefficient and partial likelihood deviance of cuproptosis-related prognostic signature in ICGC cohort (Fig. 5A and B). The risk score of STAD cases, survival status, and gene expression of verification prognostic signature in ICGC cohort was shown in Figure 5C. As expected, the data also demonstrated a poor OS rate in STAD patients with high-risk score (Fig. 5D, *P* = .044, HR = 4.9), with an AUC of 0.759 and 0.726 in a 3-year and 5-year ROC curve (Fig. 5E).

### 3.4. Immune infiltration and drug sensitivity analysis

**Figure 6.**
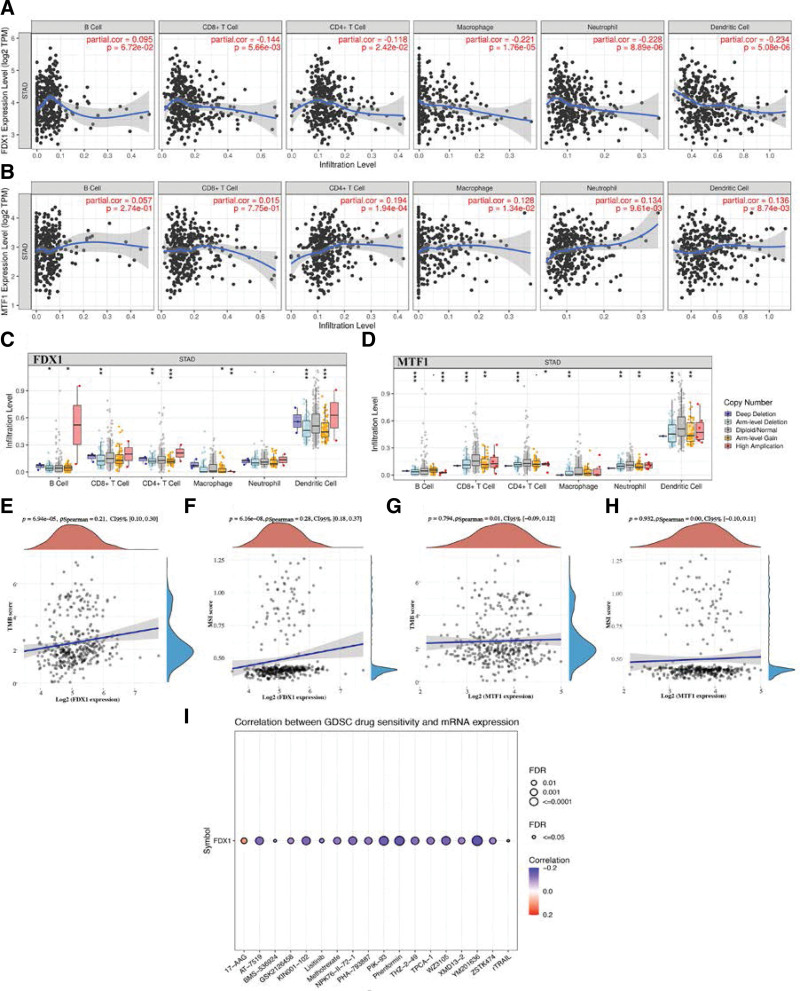
Immune cell infiltration, TMB, MSI and drug sensitivity analysis of FDX1/MTF1 in STAD. (A-B) Correlation between the expression of FDX1 and MTF1 and the abundance of immune cell in STAD. (C-D) Correlation between CNV of FDX1 and MTF1 and immune cell infiltration in STAD. (E-F) Correlation between FDX1expression and TMB/MSI score in STAD. (G-H) Correlation between MTF1 expression and TMB/MSI score in STAD. (I) Correlation between MTF1 expression and drug sensitivity in STAD. MSI = microsatellite instability, STAD = stomach adenocarcinoma, TMB = tumor mutational burden.

As shown in Figure 6A, the data demonstrated a negative correlation between FDX1 expression and the abundance of CD8 + T cell, CD4 + T cell, Macrophage, Neutrophil and Dendritic cell (Fig. 6A, all *P* < .05). Moreover, MTF1 expression increased as the immune infiltration level of CD4 + T cell, Macrophage, Neutrophil and Dendritic cell increased (Fig. 6B, all *P* < .05). Further analysis revealed that somatic cell copy number alteration of cuproptosis-related prognostic signature could inhibit the infiltration level of some immune cells (Fig. 6C and D). We also analyzed the correlation between cuproptosis-related prognostic signature and TMB/MSI score. Interestingly, the TMB (Fig. 6E, *P* = 6.94e^-5^) and MSI (Fig. 6F, *P* = 6.16e^-8^) score in STAD increased as FDX1 expression increased. However, there is no significant correlation between MTF1 expression and TMB (Fig. 6G) and MSI (Fig. 6H) score (*P* > .05). To identify a drug therapy target, a crucial way is clarifying the correlation between gene and exited drug. In our study, drug sensitivity analysis revealed that low FDX1 were positively correlated with drug resistance of genomics of drug sensitivity in cancer (Fig. 6I). Thus, FDX1 may play a vital role in the progression of STAD, and we selected FDX1 for further analysis.

### 3.5. Development of a lncRNA-miRNA-mRNA regulatory axis

**Figure 7.**
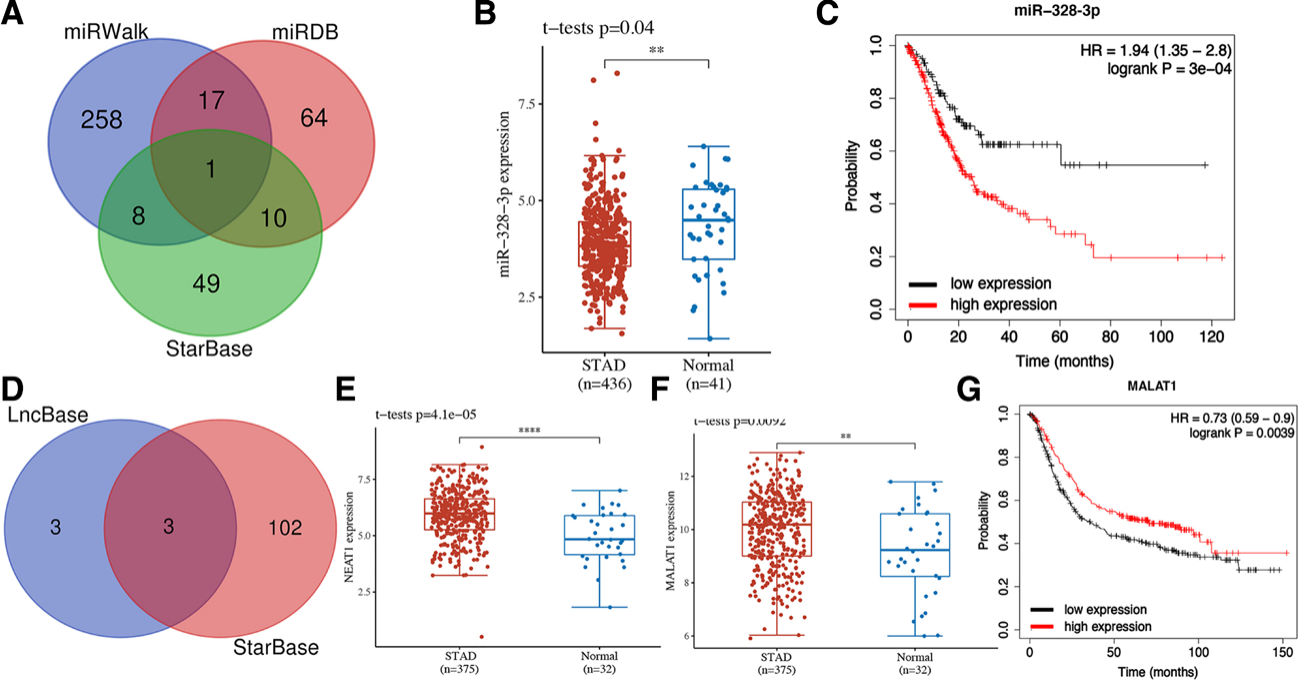
lncRNA-miRNA-mRNA regulatory axis in STAD. (A) The miRNA targets predicted by miRDB, miRWalk, and StarBase. (B and C) The expression and prognosis value of miR-328-3p in STAD. (D) The lncRNA targets predicted by lncBase and StarBase. (E and F) The expression of NEAT1 and MALAT1 in STAD. (G) The prognosis significance of MALAT1 in STAD. STAD = stomach adenocarcinoma.

We finally conducted a lncRNA-miRNA-mRNA regulatory axis analysis to further clarify FDX1 associated mechanism in STAD. For miRNA targets, miR-328-3p was identified as the miRNA target of FDX1 (Fig. 7A). Interestingly, further analysis revealed that miR-328-3p expression was downregulated in STAD (Fig. 7B, *P* = .04) and STAD patients with high miR-328-3p expression had a poor OS rate (Fig. 7C, *P* = 3e^-4^). For lncRNA targets, 3 miRNAs (NEAT1 MALAT1 and AL355075.4) were identified as the lncRNA targets of miR-328-3p (Fig. 7D). Among these 3 lncRNAs, lncRNA NEAT1 and MALAT1 were upregulated in STAD (Fig. 7E and F) and STAD patients with high MALAT1 level had a better survival (Fig. 7G, *P* = .0039), suggesting MALAT1 as most promising lncRNA target of miR-328-3p. Thus, lncRNA MALAT1/ miR-328-3p/FDX1 regulatory axis may play a vital role in the progression in STAD.

## 4. Discussion

Cu is an indispensable trace element associated with many biological processes. Excess intracellular Cu could induce proteotoxic stress and result in a new form of mitochondrial cell death termed cuproptosis, which was different from apoptosis, ferroptosis, and necroptosis.^[5,14]^ Increasing evidence revealed that the Cu levels of cancer patients are significantly elevated both in serum and tumor tissues compared to healthy people.^[15]^ Interestingly, previous studies suggested that cuproptosis-related signature or scoring model had a good performance in predicting the clinical outcome and therapy response in certain types of cancer, including pancreatic carcinoma and glioma.^[16,17]^ However, the role of cuproptosis-related genes in STAD had not been fully clarified. In our study, we systematically studied the role of cuproptosis-related genes in STAD.

Expression analysis revealed that the expression of FDX1, LIPT1, DLD, DLAT, PDHA1, PDHB, MTF1, GLS, and CDKN2A was upregulated in STAD versus normal tissue. Further analysis demonstrated that cuproptosis-related genes were mainly associated with tricarboxyic acid cycle, pyruvate metabolic process, glucose metabolic process, mitochondrial matrix, metabolic pathways, TCA cycle, glucagon signaling pathway, HIF-1 signaling pathway, and lipoic acid metabolism. HIF-1α is the crucial factor adapted to tumor hypoxia. HIF-1 signaling pathway was involved in metabolism, inflammation, vascular homeostasis and tumorigenesis under hypoxia conditions.^[18]^ TCA cycle play a vital role in energy production and macromolecule synthesis of oncogene and tumor suppressor expression.^[19]^

Further analysis demonstrated that STAD patients with high expression of CDKN2A, DLD, GLS, and MTF1 and low expression of DLAT, FDX1, PDHA1, and PDHB had a poor OS and PPS rate, demonstrating CDKN2A, DLD, GLS, MTF1, DLAT, FDX1, PDHA1, and PDHB as potential biomarkers for STAD. Interestingly, previous suggested these genes as biomarkers for the diagnosis for other types of cancers. MTF1 was suggested as a potential marker for early diagnosis and target therapy in ovarian cancer,.^[20]^ In hepatocellular carcinoma, CDKN2A was a biomarker predicting the prognosis and immune infiltrates.^[21]^ Moreover, DLAT was found to be a potential biomarker for colon adenocarcinoma.^[22]^

We then developed a cuproptosis-related prognostic signature containing 2 genes (FDX1 and MTF1) for STAD, which predicted the OS rate with medium to high accuracy. As far as we know, this is the first cuproptosis-related prognostic signature identified in human cancer. There is doubt that many prognostic signatures had been identified for STAD. Wang et al identified a necroptosis-related prognostic signature STAD by performing bioinformatics analysis.^[23]^ Another study developed a prognosis signature containing 14 lncRNAs could predict OS of STAD.^[24]^

Another important finding of our study was that we identified a lncRNA MALAT1/miR-328-3p/FDX1 regulatory axis for the progression of STAD. Previous studies revealed that lncRNA MALAT1 could accelerate tumor proliferation, migration and invasion in STAD.^[25]^ Moreover, MALAT1 was associated with cisplatin resistance in STAD.^[26]^ Zhe et al found that miRNA-328-3p could promote the progression of STAD.^[27]^ FDX1 was suggested as a prognosis biomarker and regulated biological processes in lung adenocarcinoma.^[28]^ In our study, the data suggested that FDX1 expression was upregulated in STAD and STAD patients with high FDX1 expression had a better OS rate. These results may that FDX1 acts as a tumor suppressor gene in STAD. Interestingly, similar results were obtained in RCC. The FDX1 expression in increased in RCC and the high expression of FDX1 was significantly correlated the well OS rate.^[29]^ LncRNA MALAT1/miR-328-3p/FDX1 regulatory axis may also play a vital role in the progression of STAD.

There are some limitations in our study. It would be better to verify the expression and prognosis of cuproptosis-related signature using clinical tissues. Moreover, lncRNA MALAT1/miR-328-3p/FDX1 regulatory axis should be verified using in vivo and in vitro experiments

## 5. Conclusion

In conclusion, multi-omics approaches were performed to develop a cuproptosis-related signature with 2 genes (FDX1 and MTF1) for STAD. We also identified a lncRNA MALAT1/miR-328-3p/FDX1 regulatory axis for STAD. Further study should be performed to verify our result.

## Author contributions

**Data curation:** Yunsheng Qin.

**Investigation:** Dongxiao Ding.

**Project administration:** Yunsheng Qin.

**Resources:** Dianqian Wang.

**Software:** Dianqian Wang.

**Validation:** Yunsheng Qin.

**Writing – original draft:** Dongxiao Ding.

**Writing – review & editing:** Yunsheng Qin.
